# Marie Curie (1867–1934): Twice Nobel Laureate and Her Enduring Legacy in Radiation Medicine

**DOI:** 10.7759/cureus.66703

**Published:** 2024-08-12

**Authors:** Nandan M Shanbhag, Abdulrahman Bin Sumaida, Khalid Balaraj

**Affiliations:** 1 Oncology/Palliative Care, Tawam Hospital, Al Ain, ARE; 2 Oncology/Radiation Oncology, Tawam Hospital, Al Ain, ARE; 3 Internal Medicine, College of Medicine and Health Sciences, United Arab Emirates University, Al Ain, ARE

**Keywords:** historical review, scientific mentorship, medical pioneers, radium, polonium, cancer treatment, nobel laureate, radioactivity, radiology, historical vignette

## Abstract

Marie Curie, a distinguished physicist and chemist, profoundly transformed the fields of radiology and medicine through her pioneering research on radioactivity. As the first woman to win a Nobel Prize and the only person to win in two different scientific fields, Physics (1903) and Chemistry (1911), Curie's achievements have left an indelible mark on medical science. This historical vignette explores her groundbreaking discoveries, including the isolation of radium and polonium, and her innovative applications of radioactivity in medicine, particularly in the treatment of cancer. It also delves into her relentless pursuit of knowledge and her role as a mentor, which inspired future generations of scientists and medical professionals. By examining Curie's contributions and enduring legacy, this article underscores her pivotal role in shaping modern medical practices and highlights her lasting influence on human health. Through this exploration, we aim to celebrate the life and achievements of a true pioneer whose work continues to inspire and drive advancements in medical science today.

## Introduction and background

Marie Curie, born Maria Skłodowska in Warsaw, Poland, in 1867, stands as a towering figure in the annals of science and medicine. Her groundbreaking work in the field of radioactivity not only earned her two Nobel Prizes but also laid the foundation for modern radiological practices and cancer treatments. This article delves into Curie's illustrious career, exploring her seminal discoveries, the impact of her research on medical science, and her enduring legacy as a mentor and pioneer.

Curie's journey began in a period when women faced significant barriers in science. Despite these challenges, she pursued her education fervently, eventually moving to Paris to study at the Sorbonne. There, she met and married Pierre Curie, and together they embarked on research that would change the world. In 1898, the Curies discovered the elements polonium and radium, a feat that earned them international acclaim and the 1903 Nobel Prize in Physics, shared with Henri Becquerel [1]. Marie Curie's subsequent isolation of pure radium in 1910 further solidified her reputation, culminating in a second Nobel Prize, this time in Chemistry, in 1911 [2].

Curie's contributions extended beyond theoretical research; she played a pivotal role in applying her discoveries to medicine. During World War I, she developed mobile radiography units, known as "Little Curies," which brought X-ray diagnostics to the battlefield, significantly improving the treatment of wounded soldiers [3]. Her pioneering work in radioactivity also led to the development of radium therapy for cancer, marking the beginning of modern oncology [4].

Moreover, Curie's legacy includes her dedication to mentoring future scientists. She was the first female professor at the University of Paris, where she inspired many students with her passion and perseverance. Her daughter, Irène Joliot-Curie, followed in her footsteps, winning a Nobel Prize in Chemistry in 1935, thus continuing the Curie legacy [5].

In examining the life and achievements of Marie Curie, we gain insight into the profound impact of her work on medical science and the enduring inspiration she provided to generations of scientists and healthcare professionals.

## Review

Early life and education

Marie Curie, born Maria Salomea Skłodowska on November 7, 1867, in Warsaw, Poland, grew up in a family that highly valued education. Her father, Władysław Skłodowski, was a mathematics and physics instructor, while her mother, Bronisława, managed a prestigious boarding school for girls. Despite financial difficulties and personal tragedies, including the deaths of her mother and sister, Curie excelled in her studies, demonstrating an early aptitude for science and mathematics [6].

In a time when higher education for women was not readily accessible in Poland, Curie joined the clandestine "Flying University," a progressive institution that operated in defiance of the Russian authorities who controlled Poland. This underground education network allowed her to continue her studies in science and philosophy. Determined to pursue a formal education, Curie moved to Paris in 1891 to study at the Sorbonne, where she lived in modest conditions while fully immersing herself in her academic work [7].

**Figure 1:**
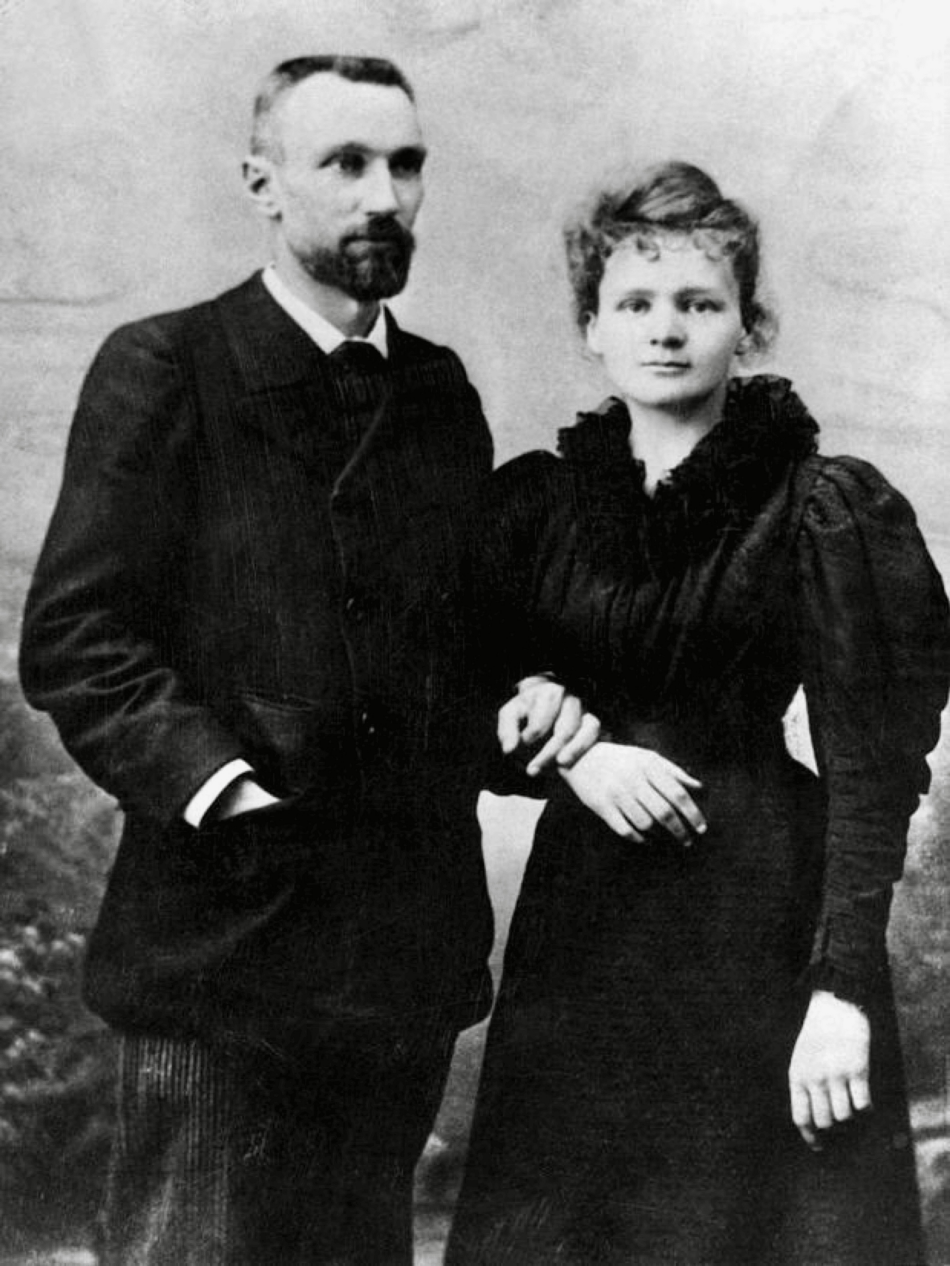
Pierre and Marie Skłodowska Curie Date: Circa 1895 Source: Wikipedia File: Pierre Curie et Marie Skłodowska Curie, 1895 [9] Author: Unknown author Permission: Public domain This work is in the public domain in its country of origin and other countries and areas where the copyright term is the author's life plus 70 years or fewer to the library. There are no known copyright restrictions on the use of this work.

At the Sorbonne, Curie earned her degree in physics in 1893, graduating first in her class. She continued her studies, obtaining a second degree in mathematics the following year. During this period, she met Pierre Curie, a distinguished physicist, who shared her passion for scientific discovery. Their mutual respect and intellectual partnership blossomed into a deep personal and professional relationship, culminating in their marriage in 1895 (Figure 1) [8,9]. Their partnership was marked by a rigorous approach to scientific inquiry and a relentless pursuit of knowledge, leading to significant breakthroughs in the understanding of radioactive elements.

The early life and education of Marie Curie are testaments to her extraordinary perseverance and dedication to science. Overcoming numerous obstacles, she laid the foundation for a career that would revolutionize the fields of physics and chemistry, ultimately transforming medical practices and advancing human health.

Discovery of radioactivity

In 1896, Henri Becquerel's discovery of natural radioactivity provided the impetus for Curie's groundbreaking research [10]. Curie coined the term "radioactivity" to describe the phenomenon and conducted exhaustive studies to measure the radioactivity of various substances [11]. Through meticulous experimentation, she discovered that thorium compounds exhibited radioactivity similar to uranium, suggesting the presence of other radioactive elements.

In collaboration with Pierre Curie, Marie Curie investigated pitchblende, a uranium-rich mineral. Their efforts led to the isolation of two new elements in 1898: polonium, named after Marie's homeland Poland, and radium [12]. These discoveries were monumental, establishing the Curies' reputation in the scientific community and earning them the 1903 Nobel Prize in Physics, shared with Henri Becquerel for their combined work on radioactivity [13].

Isolation of pure radium

Marie's continued research focused on isolating pure radium, a task fraught with difficulty due to the element's highly radioactive nature and scarcity. In 1910, she succeeded, further demonstrating her extraordinary perseverance and scientific acumen. This achievement not only solidified her scientific legacy but also had profound implications for medical science, particularly in the development of radium therapy for cancer treatment.

**Figure 2:**
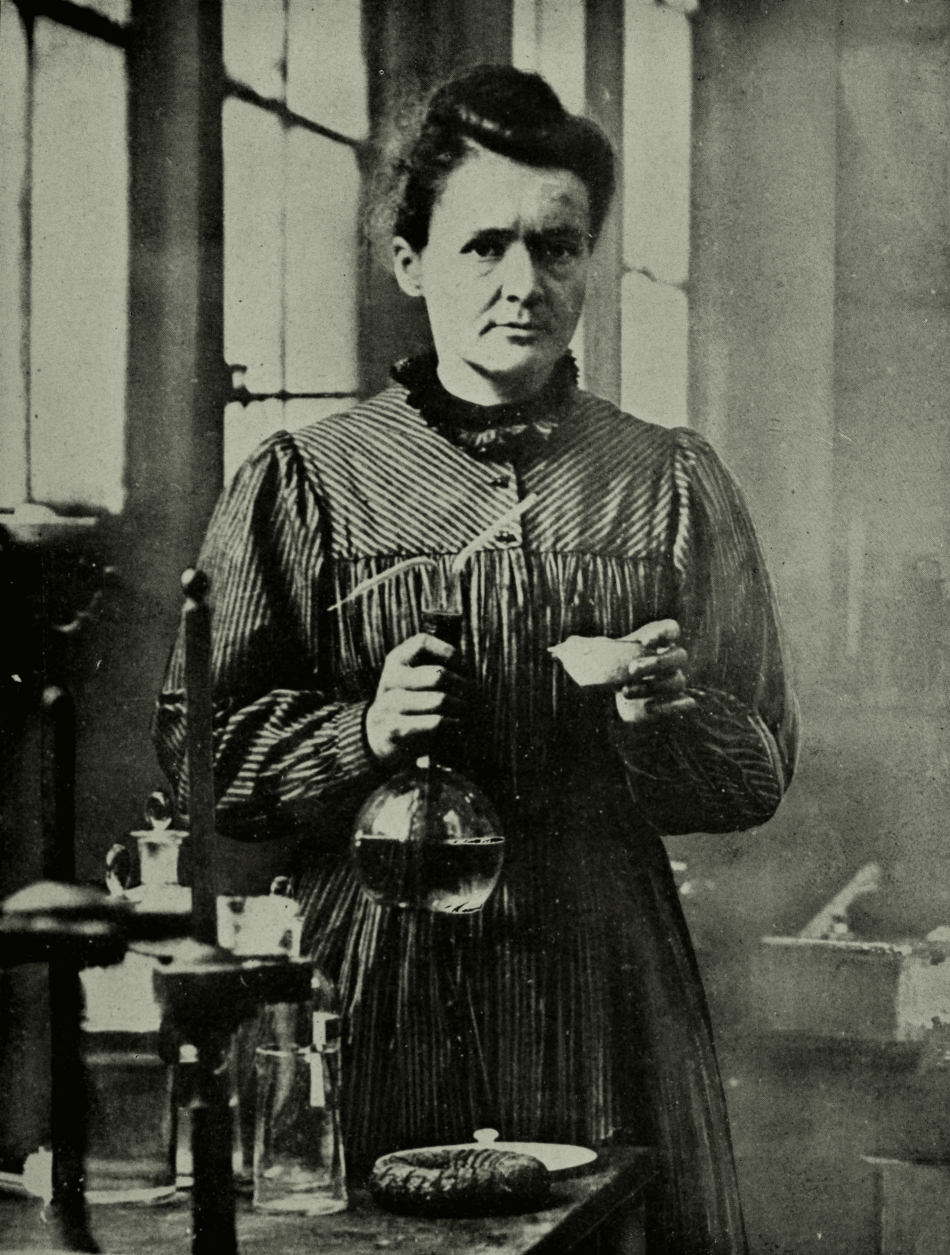
Marie Curie in her lab Date: Circa 1921 Source: The World's Work 16, 525 (1921) and Scientific Monthly 12, 580 (1921) [14] Author: "Wide World Photos" and "Underwood and Underwood, New York" This work is from the Library of Congress. According to the library, there are no known copyright restrictions on the use of this work. Images submitted for copyright by Underwood & Underwood are in the public domain in the United States due to expiration or lack of renewal.

In 1911, Marie Curie received her second Nobel Prize, this time in Chemistry, for her discovery of radium and polonium and her investigation of their properties. This remarkable achievement made her the first person to win Nobel Prizes in two different scientific fields. Her work laid the foundation for future research in nuclear physics and chemistry, profoundly influencing the scientific community (Figure 2) [14].

Contributions to medicine

Marie Curie's work transcended the laboratory, significantly impacting medical practice. During World War I, she recognized the potential of X-rays to aid in battlefield medical care. She developed mobile radiography units, or "Little Curies," which were used to provide immediate diagnostic capabilities to surgeons at the front lines. These units played a critical role in improving the survival and recovery rates of wounded soldiers [15].

Curie's pioneering efforts in radiotherapy marked the inception of oncological treatments using radioactive elements. The application of radium in treating tumors provided a new avenue for cancer therapy, one that continues to evolve today. Her work laid the groundwork for modern radiation therapy, a cornerstone of contemporary cancer treatment [16].

Mentorship and legacy

Beyond her scientific achievements, Curie's role as an educator and mentor had a lasting impact on future generations of scientists. She was the first woman to become a professor at the University of Paris, where she inspired countless students with her rigorous approach to research and her dedication to scientific inquiry [17]. Her daughter, Irène Joliot-Curie, followed in her footsteps, contributing to the family's scientific legacy by winning a Nobel Prize in Chemistry in 1935 [18].

Controversies and recovery

Her career was not without controversies. One major controversy involved her relationship with Paul Langevin, a fellow physicist, which caused significant public scandal and scrutiny. This scandal was exacerbated by the prevailing social norms of the time, which were particularly harsh on women in science [19].

Another controversy arose from the recognition and credit for scientific discoveries. While Curie received two Nobel Prizes, there were debates about the extent to which her contributions were overshadowed by those of her male colleagues, including her husband Pierre Curie and Henri Becquerel. This issue highlights the broader historical challenge of women scientists receiving due recognition for their work [20].

Additionally, there were health controversies related to her research. Curie's extensive work with radioactive materials, without the knowledge of their harmful effects, led to her own health decline. Her research practices raised concerns about the safety protocols in scientific research, which were not well established at the time [21].

Martyr to science

Marie Curie passed away on July 4, 1934, at the age of 66, at the Sancellemoz Sanatorium in Passy, France. Her death was attributed to aplastic anemia, a condition resulting from prolonged exposure to radiation due to her scientific research [22]. She was survived by her two daughters, Irène, born in 1898, and Eve, born in 1904.

Curie's legacy is not only measured by her scientific discoveries but also by her unwavering commitment to advancing knowledge and improving human health. Her life and work continue to inspire scientists worldwide, embodying the relentless pursuit of discovery and the profound impact of scientific innovation on society.

## Conclusions

Marie Curie's contributions to science and medicine are unparalleled. Her discovery of radioactivity, the isolation of polonium and radium, and the development of practical applications for her research have left an indelible mark on medical science. Her efforts in advancing radiological techniques and cancer therapies have saved countless lives and continue to influence modern medical practices. Curie's legacy as a scientist, mentor, and pioneer remains a testament to her extraordinary intellect and enduring dedication to the betterment of humanity.
